# Development of a patient decision aid for discharge planning of hospitalized patients with stroke

**DOI:** 10.1186/s12883-022-02679-1

**Published:** 2022-07-05

**Authors:** J. C. M. Prick, S. M. van Schaik, I. A. Deijle, R. Dahmen, P. J. A. M. Brouwers, P. H. E. Hilkens, M. M. Garvelink, N. Engels, J. W. Ankersmid, S. H. J. Keus, R. The, A. Takahashi, C. F. van Uden-Kraan, P. J. van der Wees, R. M. Van den Berg-Vos, On behalf of the Santeon VBHC STROKE group, S.M. van Schaik, P.J.A.M. Brouwers, P.H.E. Hilkens, G.W. van Dijk, R.A.R. Gons, R. Saxena, E.S. Schut

**Affiliations:** 1 Santeon, Utrecht, the Netherlands; 2 Department of Neurology, OLVG West, Jan Tooropstraat 164, 1061 AE Amsterdam, the Netherlands; 3 Department of Quality and Improvement, OLVG, Amsterdam, the Netherlands; 4 Amsterdam Rehabilitation Research Center/Reade, Amsterdam, the Netherlands; 5 Department of Neurology, Medisch Spectrum Twente, Enschede, the Netherlands; 6 Department of Neurology, St. Antonius Hospital, Nieuwegein, the Netherlands; 7 Department of Value Based Healthcare, St. Antonius Hospital, Nieuwegein, the Netherlands; 8 Department of Health Technology and Services Research, Technical Medical Center, University of Twente, Enschede, the Netherlands; 9 Development and Implementation of Decision Aids, ZorgKeuzeLab, Delft, the Netherlands; 10 Radboud University Medical Center, Radboud Institute for Health Sciences, IQ Healthcare and Department of Rehabilitation, Nijmegen, the Netherlands; 11 Amsterdam UMC, Location AMC, Department of Neurology, Amsterdam, the Netherlands; 12 Department of Neurology, Canisius Wilhelmina Hospital, Nijmegen, the Netherlands; 13 Department of Neurology, Catharina Hospital, Eindhoven, the Netherlands; 14 Department of Neurology, Maasstad Hospital, Rotterdam, the Netherlands; 15 Department of Neurology, Martini Hospital, Groningen, the Netherlands

**Keywords:** Stroke, Patient decision aid, Patient empowerment, Discharge planning, Shared decision-making, Outcome information

## Abstract

**Background:**

Patient involvement in discharge planning of patients with stroke can be accomplished by providing personalized outcome information and promoting shared decision-making. The aim of this study was to develop a patient decision aid (PtDA) for discharge planning of hospitalized patients with stroke.

**Methods:**

A convergent mixed methods design was used, starting with needs assessments among patients with stroke and health care professionals (HCPs). Results of these assessments were used to develop the PtDA with integrated outcome information in several co-creation sessions. Subsequently, acceptability and usability were tested to optimize the PtDA. Development was guided by the International Patient Decision Aids Standards (IPDAS) criteria.

**Results:**

In total, 74 patients and 111 HCPs participated in this study. A three-component PtDA was developed, consisting of:

1) a printed consultation sheet to introduce the options for discharge destinations, containing information that can be specified for each individual patient;

2) an online information and deliberation tool to support patient education and clarification of patient values, containing an integrated “patients-like-me” model with outcome information about discharge destinations;

3) a summary sheet to support actual decision-making during consultation, containing the patient’s values and preferences concerning discharge planning.

In the acceptability test, all qualifying and certifying IPDAS criteria were fulfilled. The usability test showed that patients and HCPs highly appreciated the PtDA with integrated outcome information.

**Conclusions:**

The developed PtDA was found acceptable and usable by patients and HCPs and is currently under investigation in a clinical trial to determine its effectiveness.

**Supplementary Information:**

The online version contains supplementary material available at 10.1186/s12883-022-02679-1.

## Background

Stroke is a major cause of disability and hospitalization worldwide [1, 2], with an estimated number of admissions of 204 per 100.000 individuals a year and an average length of hospital stay of 6.2 days [3, 4]. For discharge planning, functional (in)dependence at admission, cognitive function, marital status, and stroke severity are important determinants [5–9]. These determinants are taken into account by health care professionals (HCPs) to determine which patients will be able to return to their home, and which patients will be transferred to either an inpatient rehabilitation facility (IRF) or to an inpatient skilled nursing facility (SNF) for rehabilitation [7]. Patient involvement in discharge planning can be accomplished by providing personalized outcome information and promoting shared decision-making (SDM).

SDM is the process in which patients and HCPs make well-informed, collaborative choices by combining the best available evidence and the patient’s values and preferences [10–13]. SDM is considered a key component of high quality care [14, 15], and can be supported with patient decision aids (PtDAs), which are evidence-based tools that address a specific health related decision, provide information about options and clarify the patient’s values and preferences [16]. PtDAs are effective tools for increasing the patient’s knowledge, reducing decisional conflict and improving patient-HCP communication [16]. The process of SDM can be further enhanced by integrating information about patient-relevant outcomes in PtDAs, which is in line with the value-based healthcare (VBHC) principles [17, 18].

For patients with stroke, several decisional support interventions have been developed [19], including several visual aids [20–22], PtDAs for reperfusion therapy [23–25], and PtDAs for secondary stroke prevention [26–31]. No PtDAs have been developed for discharge planning. In order to empower patients to participate in the decision-making process about their discharge destination, the aim of this study was to develop a PtDA with integrated outcome information for hospitalized patients with stroke.

## Methods

A detailed description of the study population, data collection, and data analysis is included in the Additional file 1. A convergent mixed methods design was used by adopting a user-centered design with patients and HCPs. This study consisted of three phases. Phase 1 comprised needs assessments among patients with stroke and HCPs using a quantitative survey, including a modified version of the Control Preference Scale (CPS) [32], and self-constructed statements about their perceptions of relevant outcome information. The patient survey also included the Decisional Conflict Scale (DCS) [33, 34]. In order to gain more in-depth insight into the data gathered in the surveys, patient focus groups were conducted. All focus groups were held according to a predetermined format and were guided to trained focus group leaders. In phase 2, the PtDA was developed in co-creation sessions with a multidisciplinary team of stakeholders (i.e., the steering group), using the International Patient Decision Aids Standards (IPDAS) criteria as a guideline [35, 36]. In phase 3, acceptability (alpha) testing was performed by a subgroup of the steering group (JP, ID & RT) by assessing the PtDA for compatibility with the minimum IPDAS criteria [36]. Usability (beta) testing was performed to optimize the PtDA, via think-aloud sessions with patients and a digital quantitative survey among HCPs that did not participate in the needs assessment or co-creation sessions.

Participating patients were recruited during their admission to the acute stroke unit in three high-volume stroke centers in different regions of the Netherlands, all of which are members of Santeon, a cooperative association of seven large teaching hospitals that use VBHC principles to continuously improve quality of care. All participating hospitals are primary stroke centers with the availability of advanced imaging techniques, personnel trained in vascular neurology, and modern acute stroke units. Participating HCPs were recruited from the Santeon hospitals, academic hospitals, non-teaching hospitals, and rehabilitation facilities. All participating patients provided written informed consent. Ethical and research governance approval was obtained from the Medical Research Ethics Committees United and the local medical ethics committees of the participating hospitals. This study was conducted from July 2019 until July 2020.

**Fig. 1:**
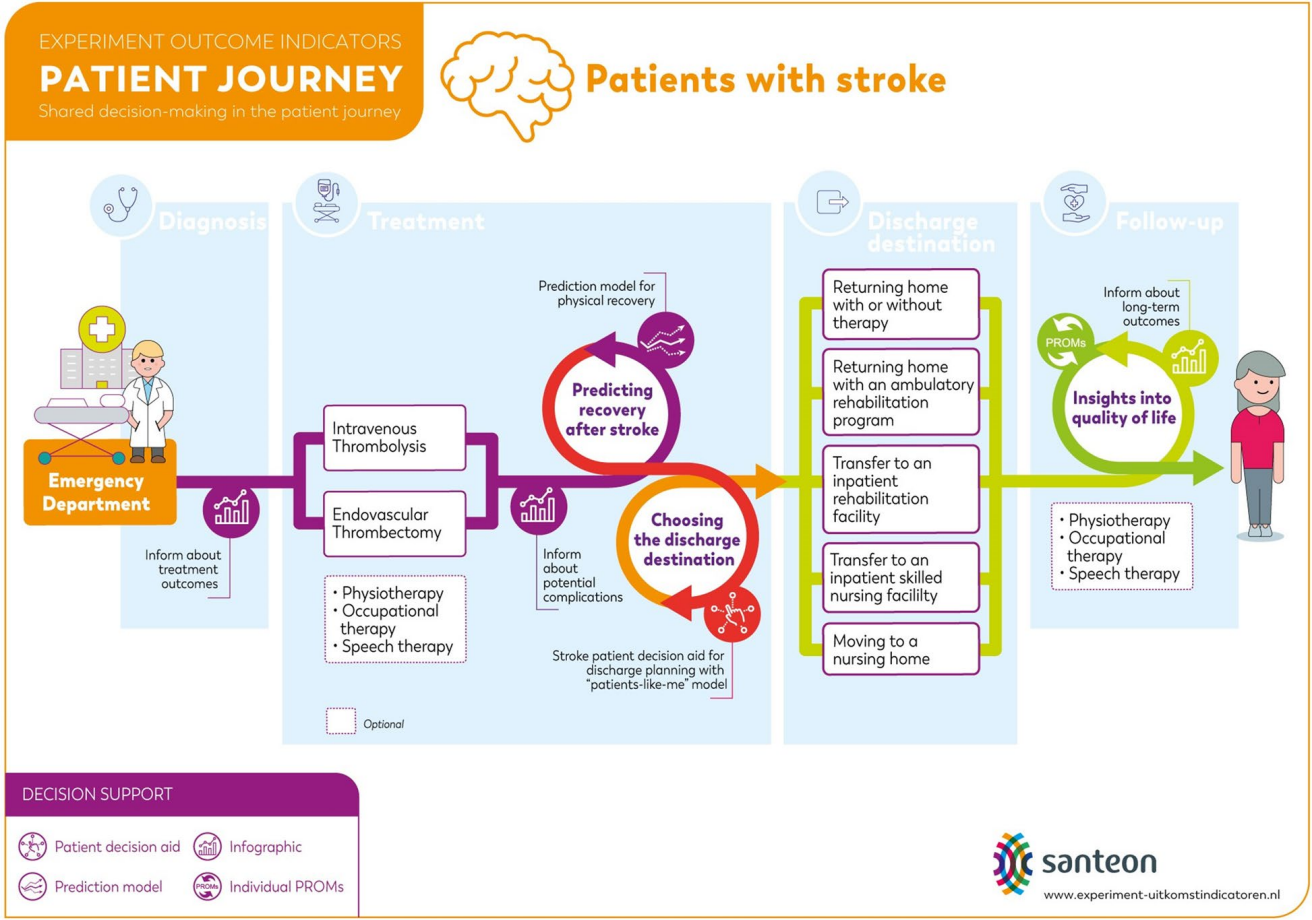
The patient journey of patients with stroke in the Netherlands. The patient decision aid for discharge planning with an integrated “patients-like-me” model supports patients with stroke and health care professionals in choosing a discharge destination

An overview of the patient journey is shown in Fig. 1, which illustrates the decision-making process about the patient’s discharge destination. A detailed description of the patient journey is included in Additional file 1. An overview of the demographic and clinical characteristics of the study population was provided using descriptive statistics. Continuous data were expressed as a mean with standard deviation (± SD), or as the median (interquartile range) where appropriate. Categorical data were expressed as frequencies (%) unless stated otherwise. Perceptions of patients and HCPs on outcome information were assessed using a 1 to 7 Likert scale, with a score of ≥ 4 points indicating relevant outcomes. Focus group discussions were audio-recorded, transcribed verbatim and coded by two independent coders (JP & NE), followed by thematic analysis. Themes related to patient perspectives on and patient preferences for decision making were identified using a combination of inductive and deductive approaches (following the framework analysis method). Quantitative data and qualitative data were analyzed with IBM SPSS (version 22) and Atlas.ti 8 for Mac.

## Results

**Fig. 2:**
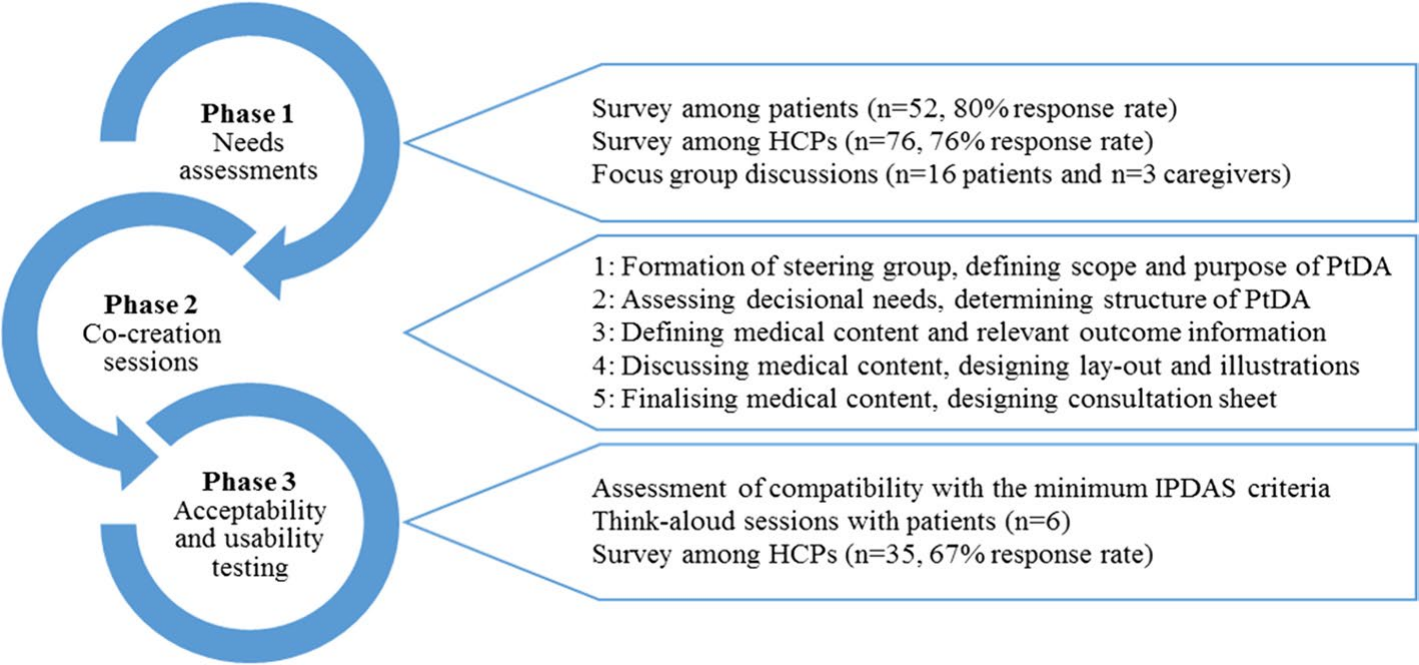
Phases of the development of the PtDA

The development process of the PtDA consisted of three consecutive phases (Fig. 2). Findings from the needs assessments were presented to the steering group and used to develop the PtDA. Findings from the acceptability and usability testing were used to optimize the PtDA.

### Phase 1: needs assessments

#### Survey among patients with stroke

Of 65 invited patients with stroke, 52 shared their decisional needs concerning discharge planning via a digital survey (response rate 80%). The mean age of the participants was 70 years, 54% were male and 42% had a low education level (all baseline characteristics are shown in Table 1). Patients reported that during admission, information concerning discharge was predominantly provided verbally (90%), and no digital information was offered. Most of the patients (60%) preferred to be actively involved in the decision-making process and the minority of the patients (31%) reported that they had been actively involved in their discharge planning. The total DCS score ranged from 0 to 81.3 (mean 58.2 ± 16.5) (Supplementary Table S1). Patients reported that the most relevant outcome information for decision-making would be information about what to expect when either returning home or being transferred to a rehabilitation facility. Also, they indicated to find it relevant to receive information about clinical outcome after one year and about patient-reported outcome measures (PROMs) of comparable patients (e.g., frequency of emotional and cognitive problems or experienced improvement in activities of daily living) (Supplementary Figure S1).

**Table 1:** Baseline characteristics of patients that participated in the needs assessment and usability test

Patient characteristic	Needs assessment (survey, *n* = 52)	Usability test (think-aloud session, *n* = 6)
Age, years – mean (SD)	70.4 (12.8)	76.6 (10.0)
Male sex	28 (53.8)	2 (33.3)
Education level		
- high	11 (21.2)	1 (16.7)
- middle	18 (34.6)	2 (33.3)
- low	22 (42.3)	1 (16.7)
- unknown	1 (1.9)	2 (33.3)
Diagnosis		
- ischemic stroke	48 (92.3)	5 (83.3)
- hemorrhagic stroke	2 (3.8)	1 (16.7)
- TIA	2 (3.8)	0 (0)
Location of ischemic stroke		
- middle cerebral artery	32 (61.5)	3 (50.0)
- anterior cerebral artery	0 (0)	0 (0)
- posterior cerebral artery	4 (7.7)	0 (0)
- vertebrobasilar	11 (21.2)	1 (16.7)
- missing	1 (1.9)	1 (16.7)
NIHSS – median (IQR)	3 (1–5)	2 (1–6)
Treatment at ED^a^		
- intravenous thrombolysis	18 (34.6)	2 (33.3)
- endovascular thrombectomy	2 (3.8)	0 (0)
- none	32 (61.5)	2 (33.3)
- missing	0 (0)	2 (33.3)
Period since stroke, months		
- 0–3 months	17 (32.7)	4 (66.7)
- 3–12 months	33 (63.5)	0 (0)
- > 12 months	1 (1.9)	2 (33.3)
- unknown	1 (1.9)	0 (0)

All data are presented as n (%) unless otherwise specified

Abbreviations: *ED* emergency department. *IQR* interquartile range, *N/A* not applicable, *NIHSS* National Institutes of Health Stroke Scale, *SD* standard deviation, *TIA* transient ischemic attack

^a^Treatment modalities were not mutually exclusive

#### Survey among health care professionals

Of 100 invited HCPs, 76 completed the digital needs assessment survey (response rate 76%). The mean age of the participants was 42 years, their average professional experience with stroke care was 12 years, and the majority worked at a teaching hospital (79%) (all baseline characteristics are shown in Table 2). The majority of the HCPs (63%) preferred active patient-involvement in the decision-making process concerning discharge, and the minority (28%) indicated that their patients are currently involved (Supplementary Table S1). According to HCPs, the most relevant information for decision-making concerning discharge is information about the patient’s circumstances at home. Also, they considered it relevant to provide information about clinical outcome after one year and about PROMs (Supplementary Figure S1).

**Table 2:** Baseline characteristics of health care professionals that participated in the needs assessment and usability test

HCP characteristic	Needs assessment (survey, n = 76)	Usability test (survey, n = 35)
Age, years – mean (SD)	42.0 (11.8)	39.6 (12.1)
Function		
- neurologist	32 (42.1)	7 (20.0)
- resident neurology	13 (17.1)	5 (14.3)
- rehabilitation specialist	8 (10.6)	1 (2.9)
- resident rehabilitation specialist	1 (1.3)	2 (5.7)
- geriatrician	0 (0)	6 (17.1)
- stroke nurse	12 (15.8)	2 (5.7)
- occupational therapist	3 (3.9)	4 (11.4)
- physiotherapist	2 (2.6)	5 (14.3)
- speech therapist	1 (1.3)	3 (8.6)
- transfer nurse	1 (1.3)	0 (0)
- other	3 (3.9)	0 (0)
Average professional experience with stroke care – mean (SD)	12.8 (10.4)	10.3 (8.3)
Organization type		
- academic hospital	2 (2.6)	5 (14.3)
- teaching hospital	60 (79.0)	22 (62.8)
- non-teaching hospital	12 (15.8)	1 (2.9)
- rehabilitation facility (IRF or SNF)	2 (2.6)	7 (20)
Self-estimated stroke knowledge		
- excellent	16 (21.1)	6 (17.2)
- good	41 (53.9)	25 (71.4)
- reasonable	18 (23.7)	4 (11.4)
- mediocre	1 (1.3)	0 (0)

All data are presented as n (%) unless otherwise specified

Abbreviations: *HCP* health care professional, *IRF* inpatient rehabilitation facility, *SNF* skilled nursing facility, *SD* standard deviation

#### Focus group discussions with patients with stroke and caregivers

In total, 16 patients with stroke and three caregivers participated in three distinct focus groups. The main topics of the focus groups were the experiences and preferences of patients with stroke and their caregivers regarding patient education, SDM, the use of (online) PtDAs, and use of outcome information in the decision-making process concerning discharge planning. Each focus group focused on a specific patient category (patients with a recent stroke, patients with a stroke more than 1 year ago, and patients with aphasia), which resulted in the identification of four themes related to the decision-making process and decisional needs concerning discharge planning:

- Provide and repeat tailored information to ensure patient education
- Present relevant outcome information in a meaningful way
- Display all information in a calm and simple manner
- Involve caregivers in decisions concerning discharge (especially for patients with aphasia)

### Phase 2: co-creation sessions

**Fig. 3:**
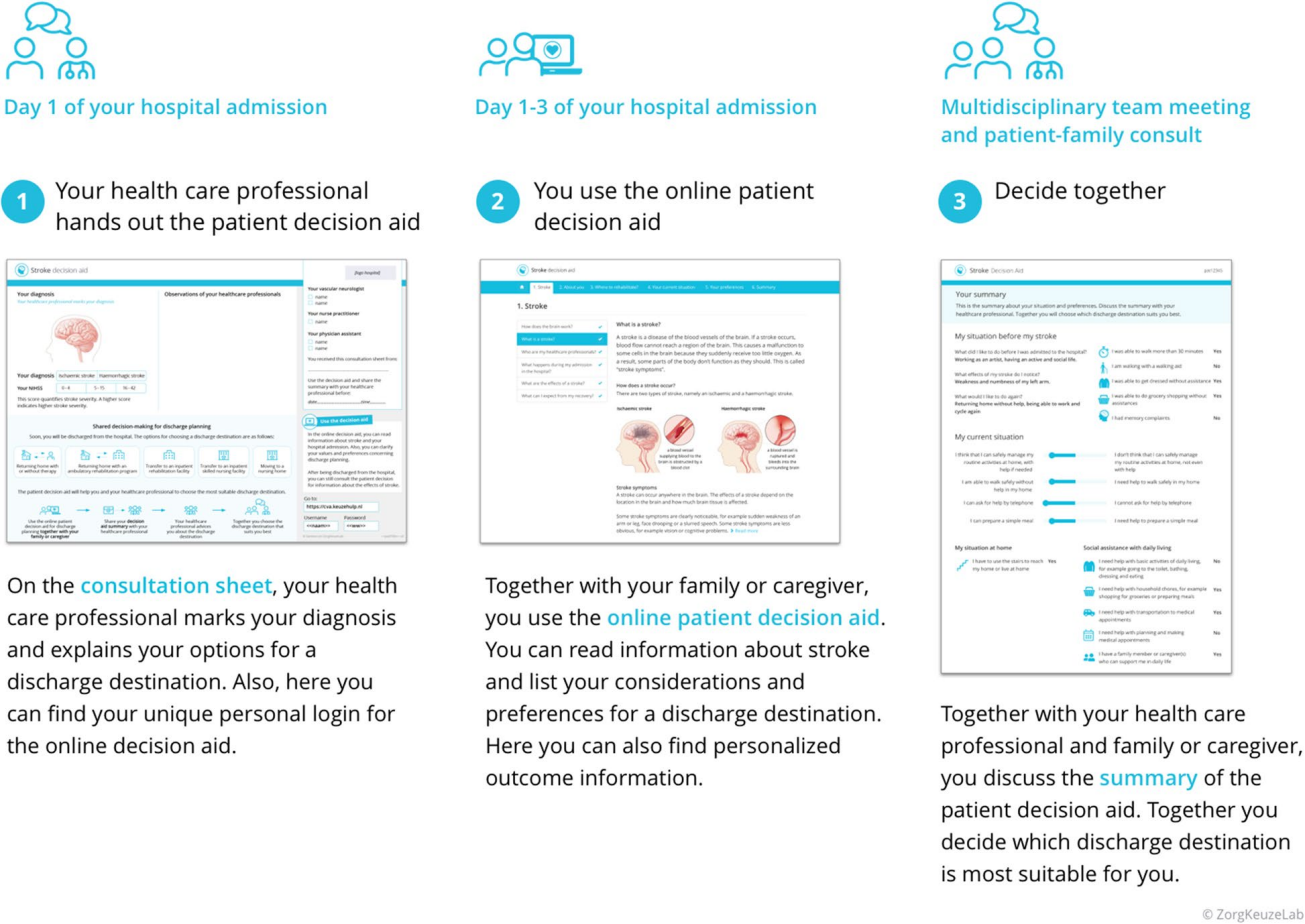
Overview of the three-component PtDA and patient instructions for using the PtDA

The steering group consisted of a patient representative, neurologists, rehabilitation specialists, geriatricians, a stroke nurse, an occupational therapist and a speech therapist. Together, they developed the PtDA in five co-creation sessions. Each session focused on a specified aspect of the PtDA (e.g., scope, structure, medical content) (Fig. 2). A three-component PtDA was developed (Fig. 3 and Supplementary Figure S2), each supporting a step in the SDM process [10, 37]:

1. a printed consultation sheet to introduce the options for discharge destinations, containing information that can be specified for each individual patient;
2. an online interactive information and deliberation tool to support patient education and clarification of patient values, containing an integrated “patients-like-me” model with personalized outcome information about the discharge destination of comparable patients with stroke. Also, a PROM questionnaire on physical and mental well-being was included;
3. a summary sheet to support actual decision-making during consultation, containing the patient’s values and preferences concerning discharge planning.

After finalizing the prototype PtDA, all content was rewritten according to the standards for creating web-based text to ensure readability and comprehensibility for all literacy groups (B1 language level according to the Common European Framework of Reference for Languages). The PtDA was acknowledged with an “easy-reading” quality mark by the Dutch foundation “Makkelijk Lezen”. For patients with aphasia, an instruction was developed that describes how to use the PtDA.

### Phase 3: acceptability and usability testing

#### Acceptability (alpha) testing

Acceptability (alpha) testing consisted of the assessment of the PtDA for compatibility with the minimal IPDAS criteria. Six qualifying criteria were considered definitional (i.e., all these criteria are required for a tool to be considered a PtDA) and six certification criteria were considered essential in order to avoid risk of harmful bias (i.e., all these criteria are required for a tool to be certified) [36]. All qualifying and certifying IPDAS criteria were fulfilled (Supplementary Table S2). In the PtDA, particular emphasis was placed on the presentation of relevant outcome information, resulting in an integrated “patients-like-me” model with personalized outcome information about the discharge destination of comparable patients with stroke.

#### Usability (beta) testing

Six patients participated in think-aloud sessions, and two of them were assisted by a caregiver. The mean age of the participating patients was 77 years and 33% were male (all baseline characteristics are shown in Table 1). By continuously verbalizing their thoughts while using the PtDA, participants mentioned that the PtDA had a user-friendly design, was easily accessible, and contained comprehensible and balanced information. All participants indicated that the textual information was too extensive and that the number of illustrations was not sufficient. Participants understood and appreciated the integrated “patients-like-me” model with personalized outcome information about the discharge destination of comparable patients with stroke. Caregiver assistance was particularly helpful for technical support and for clarification of the patient’s values.

Of 52 invited HCPs, 35 critically reviewed the PtDA and provided feedback via a digital survey (response rate 67%). The mean age of the participants was 40 years, their average professional experience with stroke care was 10 years, and the majority worked at a teaching hospital (63%) (all baseline characteristics are shown in Table 2). All respondents agreed upon the rationale behind the development of the PtDA, and the majority (86%) indicated that the PtDA contained clear, comprehensive and balanced information. Two respondents (6%) expressed concerns that the information was too complicated for patients with cognitive impairments, and for non-native speakers. One-third of the respondents (34%) indicated that the textual information was too long and suggested to remove specific parts (e.g., explanations of pre-hospital triage and procedures at the Emergency Department), to replace some parts with optional “read more” sections, and to use more illustrations. The majority (89%) appreciated the integrated “patients-like-me” model with personalized outcome information about the discharge destination of comparable patients with stroke. Several suggestions were made for including additional patient-relevant outcome information in the PtDA (e.g., clinical prognosis, recurrent stroke risk, PROMs about results at each discharge destination).

All the comments of patients and HCPs were listed. Subsequently, the aspects of the content that did not satisfy either the patients or the HCPs or both were addressed and adjusted by the steering group (Supplementary Table S3), resulting in the final version of the PtDA. 

## Discussion

The PtDA with integrated outcome information for discharge planning of hospitalized patients with stroke was developed to support patient education, clarification of patient values, and the process of SDM. During the iterative development process, relevant stakeholders were engaged in the design and testing of the PtDA. All of the minimum qualifying and certification criteria of IPDAS were met [36], and the PtDA was optimized after usability testing among patients with stroke and HCPs.

Although SDM is an increasingly promoted approach in stroke care [38], most research focuses on decision support for secondary stroke prevention [26–31], and to a lesser extent on decision support for reperfusion therapy [23–25]. Recent studies have shown that, irrespective of the chosen discharge destination, patient engagement and SDM in planning the discharge of patients with stroke can improve the patient’s knowledge and skills in coping with challenges during rehabilitation and their self-management capabilities in daily activities [39, 40]. Results from our needs assessments underline that the majority of the patients prefer to be engaged and that their decisional conflict is high. Patient-centered tools such as the PtDA developed in the present study can facilitate adequate decision support by providing personalized outcome information that is presented in a patient-friendly and balanced manner. Patients considered the integrated “patients-like-me” model useful and indicated that this information was motivating and hopeful. Future research should address whether such outcome information can be enriched, for example by integrating personalized PROM data or machine learning prediction models for discharge destination and functional independence [41, 42].

A limitation of this study was that only patients with a minor stroke were included. Although one focus group was conducted with patients with aphasia, patients with a severe stroke or other cognitive impairments could not provide input for or give feedback on the PtDA. A larger study population with a wider range of stroke severity could have enhanced the generalizability of our results. Also, other determinants for discharge planning, such as marital status and functional (in)dependence at admission, should ideally have been included in the “patients-like-me” model. However, since this information is currently not readily available, efforts should be made to prospectively collect these data in a standardized manner. Another limitation is that the accessibility of the PtDA is limited, since digital skills and Dutch language proficiency are essential for using the tool. However, since caregivers are often involved in discharge planning, they are encouraged to join and assist patients while using the PtDA. Furthermore, the PtDA will be translated to different languages when its effectiveness has been demonstrated.

## Conclusions

In conclusion, our user-centered design process resulted in an acceptable and usable PtDA with integrated outcome information to support hospitalized patients with stroke and HCPs in SDM about the discharge destination. Since any patient could benefit from consistent information provision and active involvement in the process of discharge planning, this study could be relevant for all patients with stroke that are admitted to an acute stroke unit. The PtDA is currently under investigation in a clinical trial to determine its effectiveness (Netherlands Trial Register registration ID: NL8375).

## Supplementary Information

**Additional file 1.** Supplemental methods.

**Additional file 2.** Supplemental results (supplementary tables S1-S3 and supplementary figuresS1-S2).

## Data Availability

The datasets used and/or analyzed during the current study are available from the corresponding author on reasonable request.

## Abbreviations

DCS: Decisional Conflict Scale
CPS: Control Preference Scale
HCP: Health Care Professional
IPDAS: International Patient Decision Aids Standards
IRF: Inpatient Rehabilitation Facility
PROM: Patient-Reported Outcome Measure
PtDA: Patient Decision Aid
SD: Standard Deviation
SDM: Shared Decision-Making
SNF: Skilled Nursing Facility
VBHC: Value-Based Healthcare
