# Supplementary material for: Development of a patient decision aid for discharge planning of hospitalized patients with stroke
Source: BMC Neurol. 2022 Jul 5;22:245. doi: 10.1186/s12883-022-02679-1 (PMC9254531; doi:10.1186/s12883-022-02679-1)
Supplement: Supplementary file 1 — Additional file 1. Supplemental methods. [file 12883_2022_2679_MOESM1_ESM.pdf]

Additional File 1: supplemental methods

Phase 1 (needs assessments)

Patients with stroke were consecutively included from July 2019 until December 2019 in all participating hospitals (OLVG Amsterdam, Maasstad Hospital Rotterdam and MST Enschede) during their admission at the neurology ward if they were above 18 years of age and able to complete a structured digital survey. Patients were excluded from study participation if they had insufficient Dutch language proficiency or were unable to complete the survey or interview due to cognitive impairments, global aphasia or altered consciousness.

Retrospective data were collected by means of a cross-sectional survey in consecutive patients who had a stroke within the past year in order to explore their experiences and preferences concerning discharge planning. The quantitative survey comprised 40 items, including questions regarding socio-demographic characteristics (e.g., age and education level), self-constructed questions about the patient's perceptions of the provision of relevant outcome information and of discharge planning, and two validated measures concerning decision-making. The quality of the decision-making process was estimated with the Decisional Conflict Scale (DCS) [1, 2]. This 16-item scale measures internal conflict about a certain decision on a 5-point Likert scale, ranging from 0 (strongly agree) to 4 (strongly disagree) resulting in a total score between 0 and 64. The total score was converted into a standardized score in a range from 0 to 100. A low score (<25) indicates implementing decisions, and higher scores indicate a higher decisional conflict (i.e., that the decision is less in line with the patient's personal values). Each patient's preferred role in the decision-making process was assessed with a modified version of the Control Preference Scale (CPS). The CPS consists of a single question with five response categories (A-E) about the preferred role of the patients in the decision-making process:

- Response A: "I prefer to make the decision"
- Response B: "I prefer to make the decision after seriously considering my health care professional's opinion"
- Response C: "I prefer that my health care professional and I share the responsibility for the decision making"
- Response D: "I prefer that my health care professional makes the decision after seriously considering my opinion"
- Response E: "I prefer that my health care professionals makes the decision"

In addition, HCPs were invited to complete a digital survey to explore their experiences and preferences concerning discharge planning. The quantitative survey comprised 40 items, including questions regarding socio-demographic characteristics (e.g., age, function and professional experience), the CPS and questions about relevant outcome information that were identical to the patient questionnaire. In

total, 35 statements about potentially relevant outcome information were rated both by the patients and by the HCPs on a Likert scale ranging from 1 (not relevant) to 7 (most relevant), with a score of ≥4 points indicating relevant outcomes. A top ten of relevant outcomes was composed, consisting of outcomes that were indicated as relevant by at least 75% of either the patients or the HCPs (Supplementary Figure S1). When available, information about these outcomes was included in the PtDA.

Phase 2 (co-creation sessions)

A steering group was formed that represented a national delegation of HCPs. All participants were directly involved in stroke care in various types of organizations (academic hospitals, teaching hospitals, non-teaching hospitals and rehabilitation facilities (IRF or SNF)). Patient participation was guaranteed via the attendance of a member of the patient association for stroke patients (Hersenletsel.nl). The co-creation sessions were led by a project leader (ID or SK), taking guidance from the International Patient Decision Aids Standards (IPDAS) criteria [3, 4]. During the iterative development process, the aim was to reach consensus within the complete steering group. Each step in the development process was documented and summarized at the end of each session. The co-creation sessions resulted in a prototype of the three-component PtDA:

1) a printed consultation sheet to introduce the decision, containing basic information that can be specified for each individual patient:
   - information about the diagnosis (i.e., a visual representation of the brain to use as a topic starter on stroke and associated consequences), the type of stroke (i.e., ischemic or hemorrhagic), the individual stroke severity score as measured by the National Institutes of Health Stroke Scale (NIHSS), and eligible options for discharge destination
2) an online interactive information and deliberation tool to support patient education and clarification of patient values, containing the following elements:
   - information about the etiology and impact of stroke, prognosis and discharge planning, PROM questionnaires on the patient's physical and mental condition, questionnaires to elaborate on the patient's situation prior to their admission to the hospital as well as to indicate personal treatment goals, and value clarification exercises
   - a "patients-like-me" model with personalized outcome information about the discharge destination of comparable patients with stroke. The patient can enter their type of stroke, stroke severity and age in the model, which then shows the discharge location of comparable patients
3) a summary sheet to support actual decision-making during consultation, containing the patient's values and preferences concerning discharge planning and individual PROM scores

The developed "patients-like-me" model about discharge destinations was based on anonymized retrospective data a large cohort of patients (n=5079) from three Santeon hospitals, namely OLVG, MST

and St. Antonius hospital. These data were primarily collected for quality improvement within VBHC projects and subsequently anonymized and categorized (based on diagnosis, age and stroke severity) by a data analyst from each hospital.

In the Netherlands, patients with stroke are in general not limited by demographical health care access in the decision-making process concerning discharge planning. In each region, several inpatient rehabilitation facilities and inpatient skilled nursing facilities are available. As a result, the "patients-like-me" model was defined by all distinct discharge destinations.

Phase 3 (acceptability and usability testing)

Acceptability (alpha) testing consisted of the assessment of the PtDA for compatibility with the minimal IPDAS criteria and was performed by a subgroup of the steering group (JP, ID & RT). Six qualifying criteria were considered definitional (i.e., all these criteria are required for a tool to be considered a PtDA) and six certification criteria were considered essential in order to avoid risk of harmful bias (i.e., all these criteria are required for a tool to be certified) [3]. All twelve criteria are listed in Supplementary Table S2.

Usability (beta) testing consisted of think-aloud sessions with patients, which is an effective method for evaluating the usability of digital health tools [5]. In addition, beta testing was conducted via a digital quantitative survey among HCPs that were not involved in the needs assessment. Patients with stroke were consecutively included in OLVG Amsterdam during their admission at the neurology ward if they were above 18 years of age and able to participate in a think-aloud session of approximately 30 to 60 minutes during admission. Patients were excluded from study participation if they had insufficient Dutch language proficiency or were unable to participate due to cognitive impairments, global aphasia or altered consciousness. In addition, patients that had suffered from stroke in the past were invited to participate in a think-aloud session to ensure that the perspective of stroke survivors was also considered during usability testing. These patients were recruited by the patient association Hersenletsel.nl.

HCPs were invited to provide feedback on the PtDA via a digital quantitative survey. First, they received background information on the motivation for and the creation of the PtDA. Next, participants were requested to critically review the PtDA. Subsequently, feedback was gathered via a 35-item survey, which included questions regarding socio-demographic characteristics (e.g., age, function and professional experience) and self-constructed questions and statements about the medical content, the provided outcome information and practical usability. All feedback of patients and HCPs on each component of the PtDA was listed and subsequently addressed if either patients or HCPs or both were not satisfied with the content (Supplementary Table S3).

Patient journey

Figure 1 in the manuscript shows the patient journey of patients with stroke in the Netherlands. The patient journey starts at the emergency department, where patients eligible for reperfusion therapy are treated with either intravenous thrombolysis and/or endovascular thrombectomy (either locally or after referral to a comprehensive stroke center according to the drip and ship paradigm). Immediately afterwards, patients are admitted to the acute stroke unit. During admission, a physiotherapist, an occupational therapist and a speech therapist will start with the rehabilitation process. If indicated, a rehabilitation specialist or geriatrician will be consulted as well. Based on the observations of all health care professionals, the neurologist will advise patients and their caregivers about the most suitable discharge destination (e.g., returning home or a transfer to either an inpatient rehabilitation facility or an inpatient skilled nursing facility). Several weeks after discharge, follow-up of patients will take place to evaluate their recovery, quality of life, and other patient-relevant outcomes.
