# Supplementary material for: Development of a patient decision aid for discharge planning of hospitalized patients with stroke
Source: BMC Neurol. 2022 Jul 5;22:245. doi: 10.1186/s12883-022-02679-1 (PMC9254531; doi:10.1186/s12883-022-02679-1)
Supplement: Supplementary file 2 — Additional file 2. Supplemental results (supplementary tables S1-S3 and supplementary figuresS1-S2). [file 12883_2022_2679_MOESM2_ESM.pdf]

Additional File 2: supplemental results

Supplementary table S1. Results from the needs assessments among patients and health care professionals

Item	Patients (n=52)	HCPs (n=76)
Information concerning discharge is provided to the patient using *		
- verbal information	47 (90.4)	72 (94.7)
- written information	1 (1.9)	24 (31.6)
- digital information	0 (0)	3 (3.9)
- unknown	4 (7.7)	0 (0)
Decision concerning discharge is currently made by *		
- the patient	16 (30.8)	21 (27.6)
- the caregiver(s)	2 (3.8)	0 (0)
- a neurologist	12 (23.1)	36 (47.4)
- a rehabilitation specialist	0 (0)	26 (34.2)
- a geriatrician	0 (0)	5 (6.6)
- a nurse (stroke or transfer nurse)	0 (0)	16 (21.1)
- all HCPs cooperatively	16 (30.8)	32 (42.1)
- unknown	6 (11.5)	0 (0)
Decision concerning discharge should preferably be made by		
- the patient	5 (9.6)	0 (0)
- the patient, after seriously considering the HCP’s opinion	7 (13.5)	7 (9.2)
- the patient and HCP together	19 (36.6)	41 (53.9)
- the HCP, after seriously considering the patients’ opinion	10 (19.2)	22 (29.0)
- the HCP	5 (9.6)	0 (0)
- missing	6 (11.5)	6 (7.9)
For patients and caregivers, information about discharge planning (from a professional perspective)		
- is sufficiently provided	N/A	35 (46.0)
- is sufficiently reliable	N/A	35 (46.0)
- should be available in any case about all relevant options	N/A	58 (76.3)
Total score on the decisional conflict scale		
- range	0-81.3	N/A
- mean (SD)	58.2 (16.5)	N/A
Overall satisfaction with the decision-making process		
- satisfied	41 (78.9)	32 (42.1)
- not satisfied	2 (3.8)	8 (10.5)
- neither satisfied nor not satisfied	3 (5.8)	32 (42.1)
- missing	6 (11.5)	4 (5.3)

All data are presented as n (%) unless otherwise specified.
Abbreviations: HCP: health care professional; N/A: not applicable; SD: standard deviation
** Answers were not mutually exclusive*

Supplementary table S2. IPDAS minimum qualifying and certification criteria for patient decision aids[1]

	IPDAS criterion	PtDA compatibility?
**Qualifying criteria**	1. The PtDA describes a health condition or problem for which a specific decision had to be made (index decision)	Yes
	2. The PtDA explicitly states the index decision that needs to be considered	Yes
	3. The PtDA describes the options available for the index decision	Yes
	4. The PtDA describes the positive features (benefits or advantages) of each option	Yes
	5. The PtDA describes the negative features (harm, side effects or disadvantages) of each option	Yes
	6. The PtDA describes what it is like to experience the consequences of the options (e.g., physical, psychological or social). The PtDA should help patients to clarify values	Yes
**Certification criteria**	7. The PtDA shows the negative and positive features of options with equal detail (e.g., using similar fonts, sequence, presentation of statistical information)	Yes
	8. The PtDA (or associated documentation) provides citations to the evidence selected	Yes
	9. The PtDA or associated documentation) provides a production or publication date	Yes
	10. The PtDA (or associated documentation) provides information about the update policy	Yes
	11. The PtDA provides information about the levels of uncertainty around event or outcome probabilities	Yes
	12. The PtDA (or associated documentation) provides information about the funding source used for development.	Yes

Abbreviations: IPDAS: International Patient Decision Aids Standards; PtDA patient decision aid

Supplementary table S3. Results from the usability test: comments on and changes in the PtDA

**PtDA component**	**Comments**	**P (+ C)**	**HCP**	**Change**
First impression	Good initiative	✓	✓	N/A
	User-friendly design	✓	✓	N/A
Printed consultation sheet	Clear layout, good color use	✓	✓	N/A
	All basic information represented	✓	✓	N/A
Online information tool	Easily accessible	✓		Access instruction clarified
	Balanced lay out	✓	✓	N/A
	Comprehensive information	✓	✓	N/A
	Consider literacy		✓	Readability checked
	Understandable language	✓		Quality mark “easy reading”
	Text too long	✓	✓	Shortened text
	Not enough illustrations	✓	✓	Added extra illustrations
	Incorrect job description of multidisciplinary team	✓	✓	Changed several job descriptions
	Differences between IRF and SNF not made explicit	✓		Adjusted the definitions of IRF and SNF
	Supportive quotes of other patients	✓		Added extra quotes
	Balanced presentation of options		✓	N/A
Online deliberation tool	Difficult value clarification exercises (statements and questions)		✓	Changed order and phrasing of exercises
	Lack of questions about household management	✓		Added question about household management
	Uncomfortable questions about caregivers if you don’t have any	✓		Rephrased questions about caregivers
	Hard to answer all questions and statements without family member	✓		Emphasized that PtDA should ideally be used with caregiver
“Patients-like-me” model with personalized outcome information	Reassuring to see that many other patients return to their home	✓		N/A
	Motivating and hopeful information	✓		N/A
	Colors of icons hard to distinguish	✓		Changed colors of icons
	Statistics are not interesting for an individual patient		✓	Clarified the aim of the outcome information
	Additional outcome information desirable (e.g., functional status)		✓	Will be added when outcome data is available
Summary sheet	Informative for own use	✓	✓	N/A
	Useful during consultation	✓	✓	N/A
General	Essential information for every patient with stroke	✓	✓	N/A
	Concerns about time investment for patients	✓	✓	Replaced some text to optional “read more” sections
	Concern about long-term practical use		✓	Accountability addressed during implementation
	Important to mention that the information remains available		✓	Added note about continuous availability of information

Abbreviations: C: caregiver; HCP: health care professional; IRF: inpatient rehabilitation facility; N/A: not applicable; P: patient; PtDA: patient decision aid; SNF: skilled nursing facility

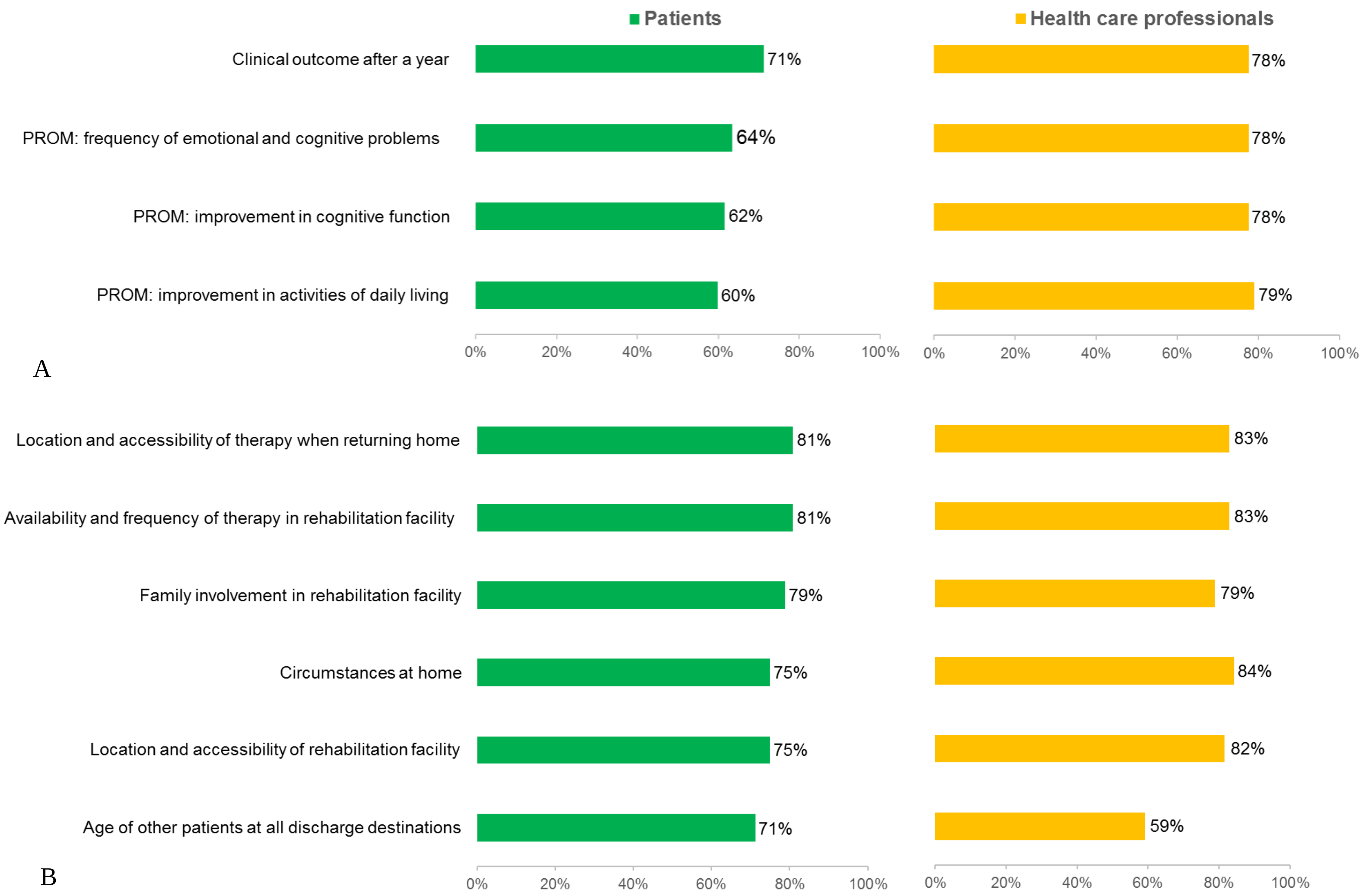

Supplementary Figure S1. Most relevant information for decision-making concerning discharge as indicated with a score of ≥4 points on a 1-7 Likert scale by patients and health care professionals.
A: information about outcomes according to patients (green) and health care professionals (yellow)
B: information about discharge destinations according to patients (green) and health care professionals (yellow)

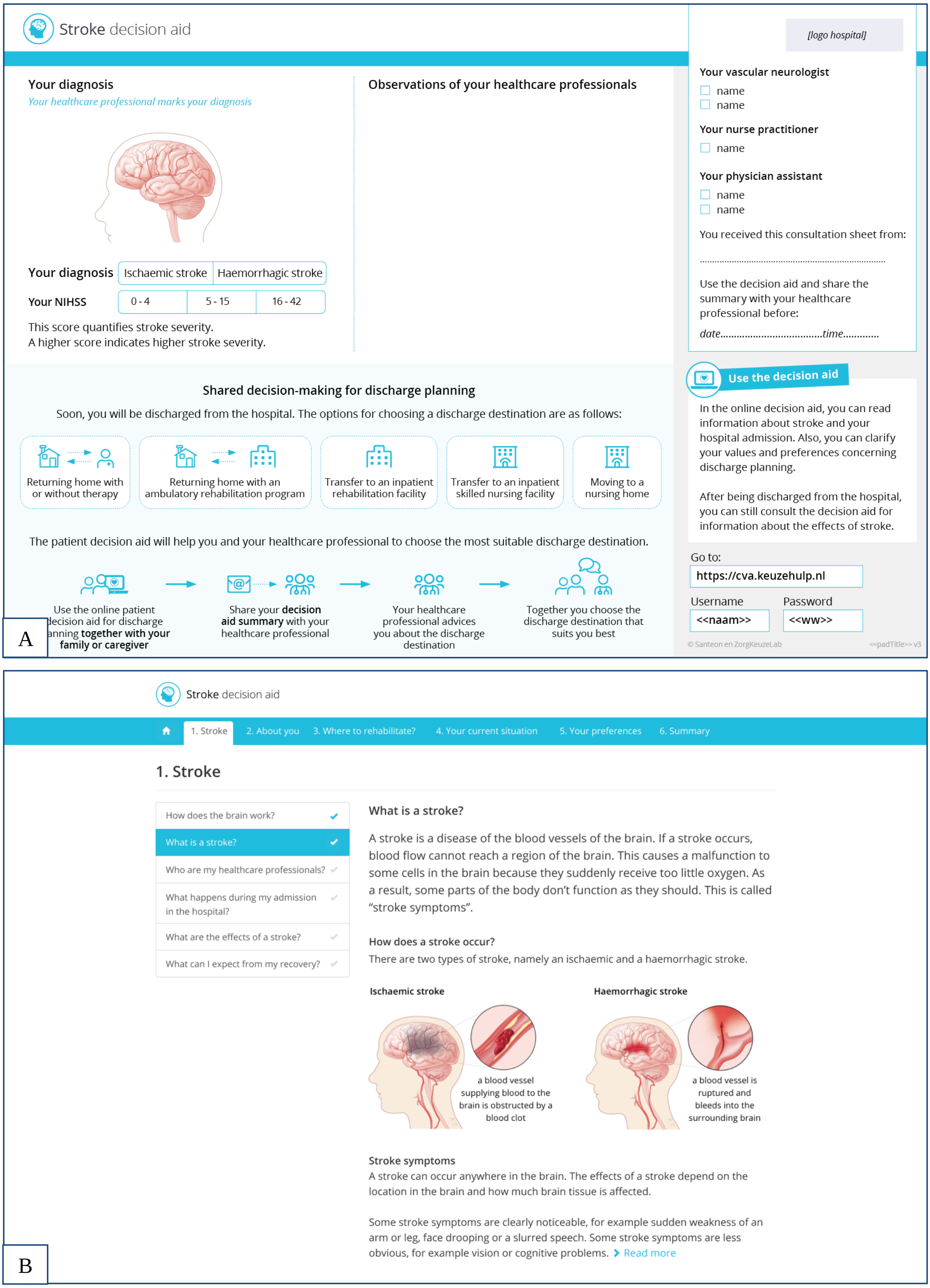

Supplementary Figure S2. Screenshots of several components of the patient decision aid
A. Printed consultation sheet
B. Online information and deliberation tool

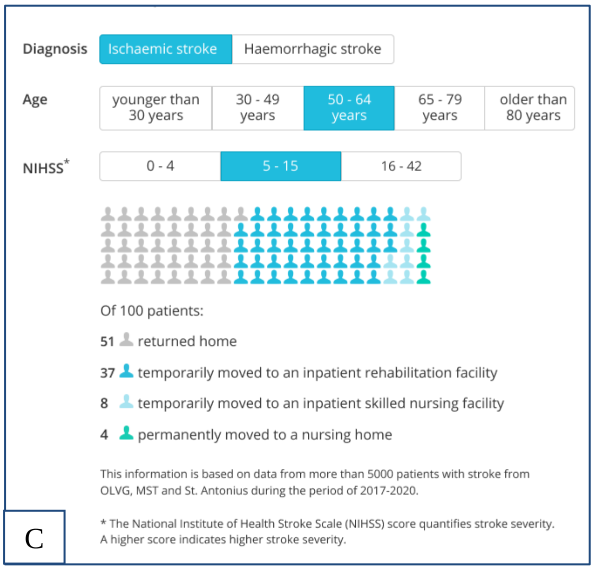

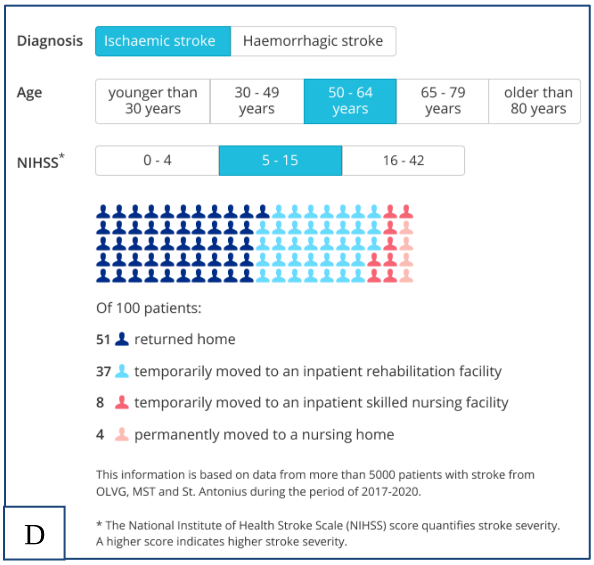

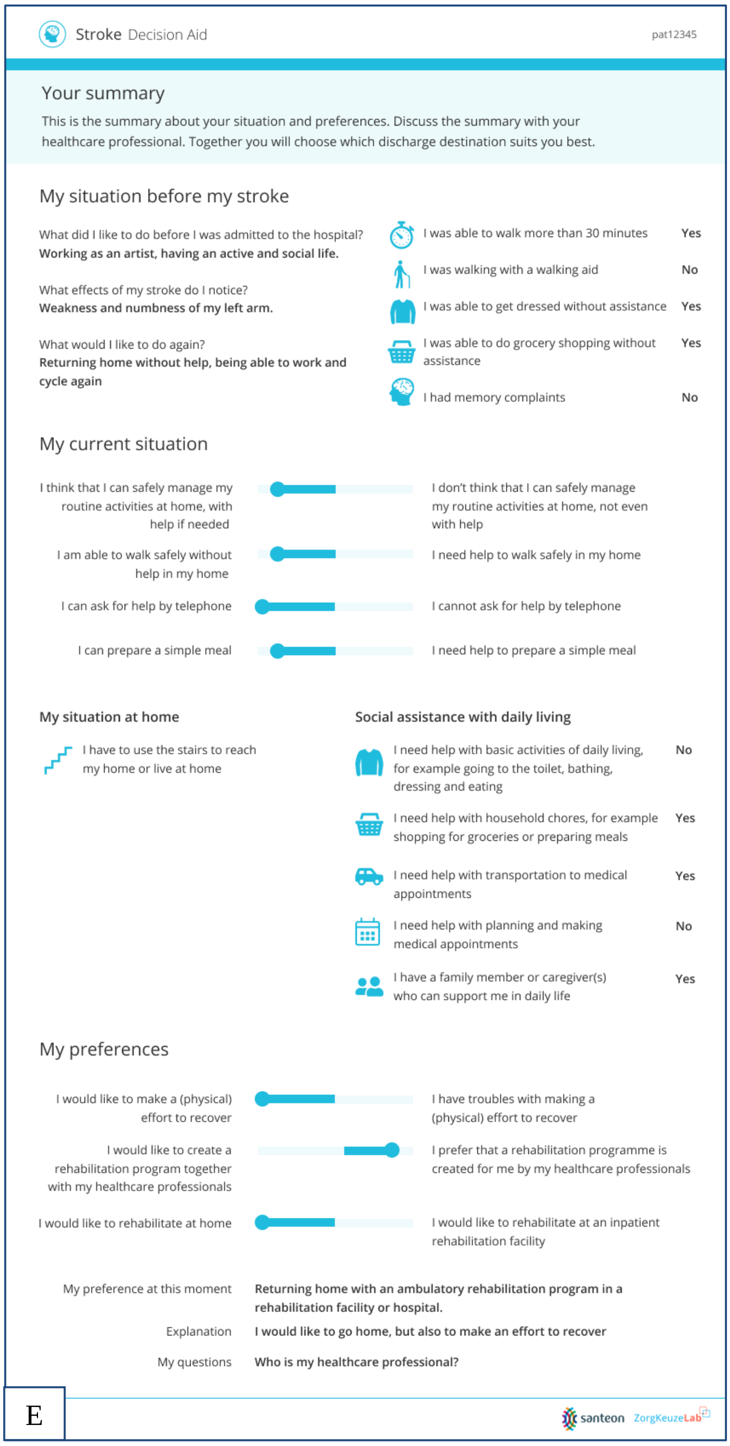

Supplementary Figure S2 (continued). Screenshots of several components of the patient decision aid
C. Integrated “patients-like-me” model with personalized outcome information
D. Different colors of the “patients-like-me” model, adjusted after usability testing
E. Summary sheet with patients’ values and preferences
